# The Spectrum of Non-ischemic Cardiac Magnetic Resonance Imaging Findings: A Retrospective Analysis

**DOI:** 10.7759/cureus.11354

**Published:** 2020-11-05

**Authors:** Talal Almas, Hassan Saleem, Maryam Ehtesham, Salman Hussain, Tarek Khedro, Reema Alsufyani, Fatimah Alahmed, Dana Almubarak, Syed Muhammad Jawad Zaidi, Aamir Hameed

**Affiliations:** 1 Internal Medicine, Royal College of Surgeons in Ireland, Dublin, IRL; 2 Radiology, Islamabad Diagnostic Center, Islamabad, PAK; 3 Internal Medicine, Rawalpindi Medical University, Rawalpindi, PAK; 4 Tissue Engineering Research Group, Department of Anatomy and Regenerative Medicine, Royal College of Surgeons in Ireland University of Medicine and Health Sciences, Dublin, IRL

**Keywords:** cardiac magnetic resonance imaging, non-ischemic cardiac pathologies

## Abstract

Introduction

Since cardiac pathologies remain ubiquitous, their prompt diagnosis through the means of innovative technologies, such as cardiac magnetic resonance imaging, remains pivotal. The spectrum of these pathologies varies widely, ranging from ischemic etiologies to rare cardiac malignancies. This study evaluates the prevalence of nonischemic cardiac pathologies, such as infiltrative heart diseases, that often warrant meticulous diagnostic evaluation through the means of cardiac magnetic resonance imaging.

Methods

We performed a retrospective study in order to analyse the cardiac magnetic resonance imaging records of 250 patients over a period of six months with previously remarkable cardiac histories. Patients with a prior history of ischemic cardiac disease, as determined from past medical and surgical records, were excluded from the study. The prevalence of various nonischemic findings was ascertained. The demographic characteristics and comorbidities of the patients were also tabulated.

Results

In the present study, 250 patients were included, of which 115 were females and 135 were males, with the mean age hovering at 48.21 ± 11.49 years. The top two most prevalent cardiac magnetic resonance imaging findings were concentric moderate-to-severe left ventricular hypertrophy and patchy subendocardial late gadolinium enhancement of the left ventricle; these were observed in 62.2% and 23.7% of the patients, respectively. Cardiac magnetic resonance imaging also divulged findings typical of rarer pathologies, including cardiac sarcoidosis and primary cardiac lymphoma.

Conclusion

Pathologies of the heart often mandate extensive diagnostic workup through the means of radiological modalities such as cardiac magnetic resonance imaging. In patients with indications of nonischemic cardiac pathologies, cardiac magnetic resonance imaging can be employed as part of the initial radiological armamentarium. Furthermore, cardiac magnetic resonance remains the imaging modality of choice for detecting infrequent cardiac pathologies, such as cardiac sarcoidosis.

## Introduction

Cardiac magnetic resonance (CMR) is a modality of cardiac imaging that has gained precedence in the field of cardiovascular imaging over the last decade due to its non-invasive nature and high sensitivity and specificity. With a single scan, high-resolution images providing an unrestricted view may be obtained. These are useful for global and regional functional assessment, particularly the determination of left ventricular ejection fraction (LVEF) and myocardium viability [1]. CMR is a versatile diagnostic tool that can be employed to distinguish ischemic and nonischemic cardiomyopathies (NICM). Additionally, late gadolinium enhancement (LGE), as witnessed during CMR procedures, can further aid in definitively elucidating the underlying etiology of a case. Specifically, CMR can be efficaciously employed in establishing the etiology of NICM cases [2,3]. In NICM, a significant derangement of the left ventricular (LV) systolic function is observed. Pertinently, the reduced LV systolic function is attributed to causes other than coronary artery disease, thus distinguishing it from other ischemic etiologies [4]. The prevalence of NICM is noted to hover at 40-50 cases per 100,000 [5]. The utilization of LGE allows clinicians to distinguish between ischemic and NICM; it can also help in differentiating the various etiologies of NICM. Although LGE imaging is very sensitive for detecting dense, focal fibrosis in nonischemic cardiomyopathy by factoring in differences in the degree of gadolinium uptake, LGE might not be able to detect findings characteristic of NICM, such as ventricular dilation, due to the myocardial signal intensity being isointense [6,7]. To this end, T1 mapping, which can divulge underlying diffuse fibrosis, can prove to be pivotal in detecting diffuse myocardial fibrosis that may otherwise go undetected with the exclusive utilisation of LGE [8]. In addition to detecting underlying myocardial fibrosis, T1 and T2 mapping are imperative in deducing other histological features seen in cardiac pathologies, such as iron overload states, sarcoidosis, and amyloidosis [9]. Due to the superiority of CMR over the majority of other imaging modalities, it has been incorporated into a multitude of current diagnostic criteria [6-9]. Nevertheless, its use is often deemed unnecessary in clinical practice, often obscuring the timely detection of the particular etiology underlying NICM. Our study describes the spectrum of findings that can be deduced from CMR imaging of patients with prior indications of nonischemic cardiac disease. The study also aims to sensitize clinicians to the usefulness of including CMR imaging among the list of initial investigations in patients with NICM.

## Materials and methods

This retrospective study involved 250 patients who demonstrated previously remarkable cardiac histories and subsequently underwent CMR imaging for the elucidation of the underlying etiologies. Patients with a prior history of ischemic diseases, such as myocardial infarction or acute coronary syndrome, were deemed ineligible for the study and were thus excluded. Moreover, patients who had undergone either coronary artery bypass grafting or percutaneous coronary intervention for ischemic pathologies were also excluded from the study. The exclusion criteria ensured that only patients with nonischemic etiologies, including infiltrative disease processes, were included in our study. The prevalence of nonischemic CMR pathologies was ascertained. The data were then analysed using the SPSS 23.0 software (Armonk, NY; IBM Corp.). Finally, the demographic characteristics and the preexisting comorbidities of the patients were also tabulated.

## Results

The current study analysed the CMR imaging records of 250 patients. The mean age of the participants hovered at 48.21 ± 11.49 years. Baseline characteristics of the study participants were considered, duly noting any obvious gender predilection. A breakdown of the participants with respect to their gender and marital status is presented in Table 1. 

**Table 1:** A breakdown of study participants with respect to their gender and marital status.

Parameters		Frequency (n)	Percentage (%)
Gender	Males	135	54%
	Females	115	46%
Marital Status	Married	224	89.6%
	Unmarried	26	10.4%

Prior to establishing the frequency of the various CMR findings, baseline patient comorbidities were established. Notably, diabetes mellitus and hypertension were the most common comorbidities observed among our study participants. Table 2 presents the prevalence of various comorbidities among patients in our study. 

**Table 2:** A breakdown of the study participants with respect to their existent comorbidities.

Comorbidities	Frequency (n)	Percentage (%)
Diabetes mellitus	87	34.8%
Hypertension	61	24.4%
Chronic kidney disease	42	16.8%
Chronic obstructive pulmonary disease	24	9.6%
Asthma	19	7.6%
No comorbidities	17	6.8%

Within the study cohort, concentric moderate-to-severe left ventricular hypertrophy and patchy subendocardial late gadolinium enhancement were the most commonly observed findings; they were observed in 62.2% and 23.7% of the patients, respectively. Interestingly, the spectrum of CMR findings included other rarer pathologies, such as primary cardiac lymphoma, which was observed in only one patient (0.4%). The detailed spectrum of the various nonischemic CMR findings is tabulated in Table 3.

**Table 3:** A delineation of the various nonischemic CMR findings. CMR: cardiac magnetic resonance.

CMR Finding	Frequency (n)	Percentage (%)
Concentric moderate-to-severe left ventricular hypertrophy	156	62.4%
Patchy subendocardial late gadolinium enhancement of ventricles	59	23.6%
Pericardial effusion	19	7.6%
Ventricular dilation	10	4.0%
Arrhythmogenic right ventricular cardiomyopathy	2	0.8%
Amyloidosis	1	0.4%
Cardiac sarcoidosis	1	0.4%
Primary cardiac lymphoma	1	0.4%
Cardiac thalassemia	1	0.4%

**Figure 1:**
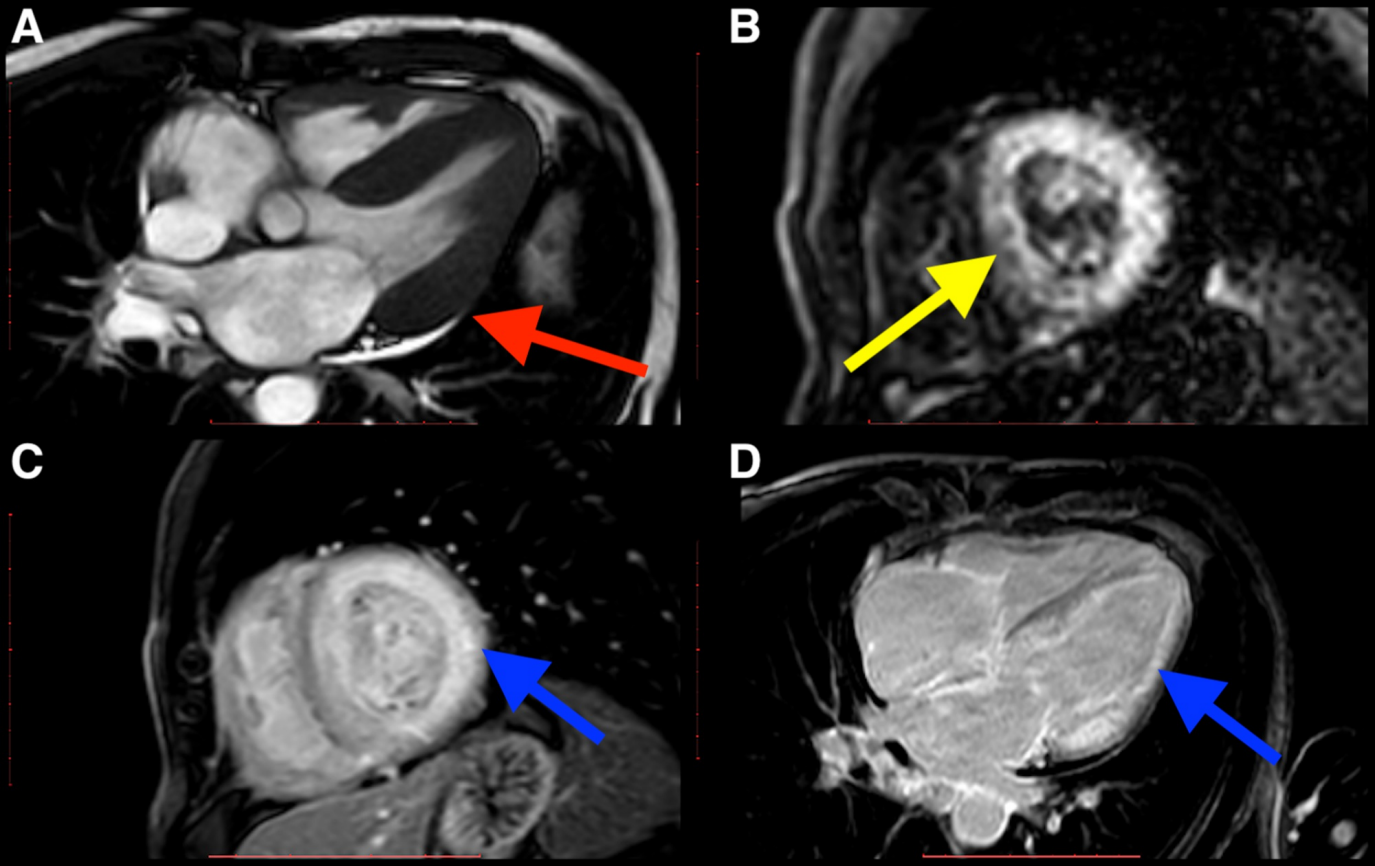
Cardiac magnetic resonance imaging of an amyloidosis patient. A four-chamber cine image depicts concentric moderate-to-severe left ventricular hypertrophy with small pericardial and bilateral pleural effusion (red arrow in A). On IR-TFE imaging, myocardial nulling could not be achieved (yellow arrow in B). In C and D, there is diffuse late gadolinium enhancement of the left ventricle, both atria and the right ventricle myocardium (blue arrows). IR-TFE images: inversion conversion-turbo field echo images.

Pertinently, findings suggestive of cardiac amyloidosis were observed in just one patient (0.4%). In this patient, the CMR findings showed that the left ventricle was normal in function with a calculated ejection fraction of around 61%. Additionally, there was evidence of moderate-to-severe left ventricular hypertrophy since both the atria were mildly dilated. The interatrial septum had also hypertrophied and diffuse LGE of the left ventricle was noted. The LGE also involved both the atria and the right ventricular myocardium. Interestingly, small pericardial and bilateral pleural effusions were also noted (Figure 1). 

**Figure 2:**
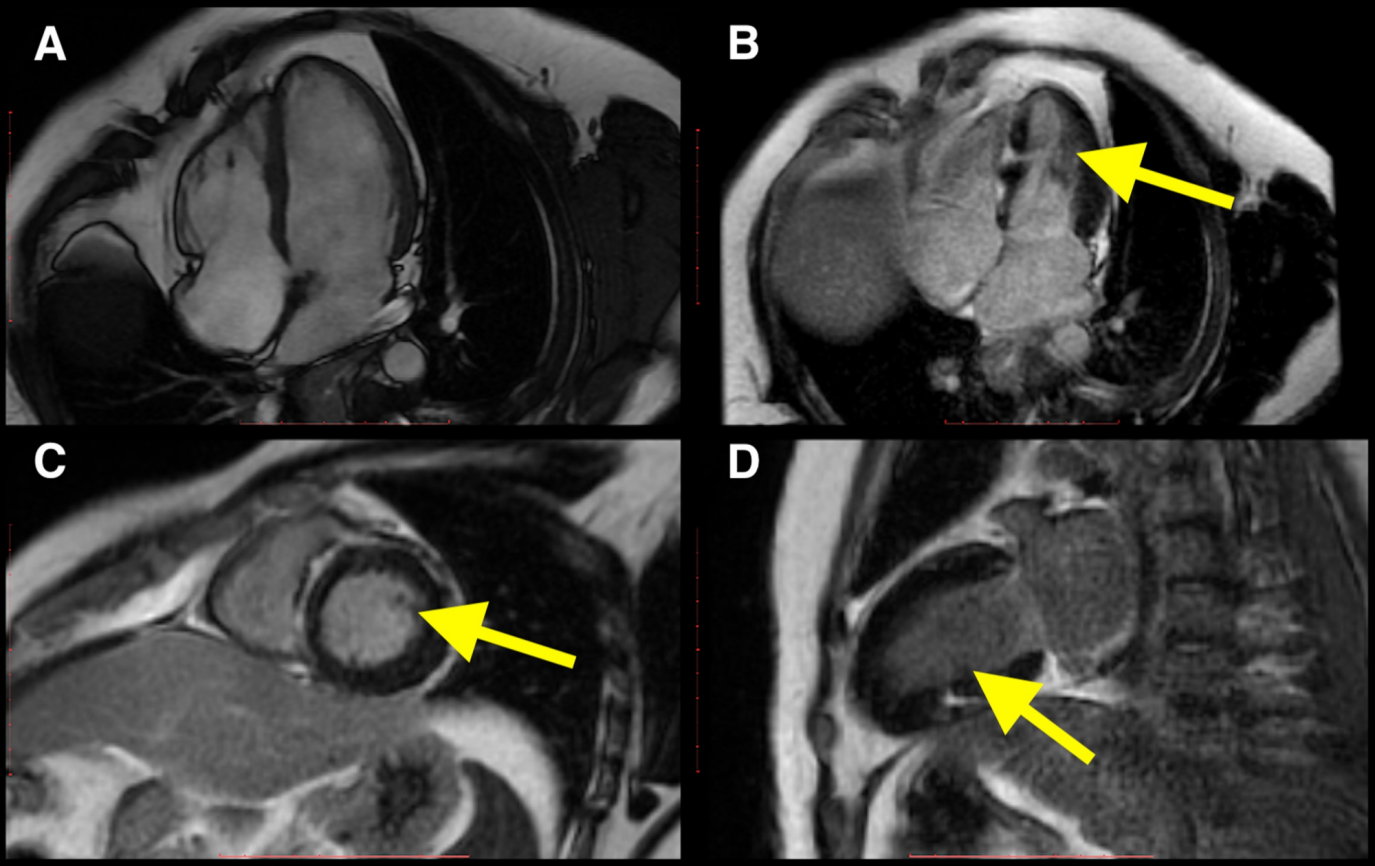
CMR imaging of a patient with cardiac sarcoidosis. Four-chamber cine imaging found no abnormality (A). There is patchy subendocardial LGE in the left ventricle (yellow arrows in B, C, and D). CMR: cardiac magnetic resonance LGE: late gadolinium enhancement

Similarly, findings characteristic of cardiac sarcoidosis were observed in just one patient (0.4% of the study participants), reaffirming the utility of CMR imaging in detecting infiltrative cardiac pathologies. In the only patient with cardiac sarcoidosis, left ventricular ejection fraction was noted to be 56 %. There was evidence of subendocardial LGE in the basal and mid-inferior septum in a patchy fashion as described above. This type of LGE can be seen in cardiac sarcoidosis and is delineated in Figure 2. 

## Discussion

Hypertrophic cardiomyopathy (HCM) is one of a diverse array of cardiomyopathies that often have nonischemic etiologies. It is thought that the ailment is hereditary in nature and is caused by mutations in sarcomere proteins that culminate in cardiac dysfunction [10]. In HCM, hypertrophy of the left ventricular wall interferes with normal cardiac function and thus forms the pathological basis of the clinical symptoms observed [11]. While a multitude of imaging modalities such as echocardiography can be employed to yield a diagnosis of HCM, in contrast to other imaging modalities, CMR allows more sensitive measurement of maximal wall thickness, which serves as an important prognostic predictor[11]. CMR has also demonstrated superiority in the detection of apical variant HCM when compared to echocardiography, which was non-diagnostic in the same patients [12,13]. Additionally, the utility of contrast-enhanced CMR in detecting myocardial fibrosis via LGE is well-established [13]. LGE in most HCM patients shows a typical pattern characterized by patchy involvement, especially at sites of septal insertion, and greatly hypertrophied ventricular walls [3]. Pertinently, the risk of sudden cardiac death increases with the extent of LGE observed [14]. A meta-analysis established that LGE, as evidenced by CMR findings, can be vital for risk stratification of HCM patients since LGE was to be correlated with odds of sudden death [10]. Pertinently, the aforesaid risk stratification is not achievable using other imaging modalities, such as echocardiography [10]. Due to its ability to improve risk stratification amongst patients with HCM, CMR is a vital diagnostic tool. 

Amyloidosis is a systemic infiltrative pathology that can afflict a myriad of organs including the heart. In both variants of amyloidosis, i.e. the immunoglobulin light chain (AL) and transthyretin (ATTR) amyloidosis, protein deposition can culminate in infiltrative disease processes that affect the heart. Cardiac amyloidosis has a poor prognosis, as patients eventually develop heart failure, angina, and arrhythmia [15]. Not only has CMR emerged as a robust imaging modality for diagnosing both types of cardiac amyloidosis, it can also distinguish between them, even though they are indistinguishable clinically [2,15]. The use of LGE CMR is useful here due to the characteristic patterns of enhancement shown by an infiltrated myocardium. In order to better distinguish between the two variants of amyloidosis, the Query amyloid late enhancement (QALE) scoring system can be employed [15,16]. The scoring system draws primarily on the differences observed in LGE within the aforesaid variants [16,17]. Notably, these characteristic LGE patterns may not be seen in some instances or may only appear late in the disease process; therefore, non-contrast characterization of amyloidotic myocardium with T1 mapping could serve as a potentially more sensitive detector of the disease in comparison to LGE [17]. In one particular study, AL amyloidosis demonstrated the highest T1 when T1 mapping was performed in 85 ATTR, 79 AL, 46 HCM, and 52 normal patients [17]. This further accentuates the clinical value of CMR in yielding improved diagnostic outcomes. 

Sarcoidosis is a systemic disease characterized by the formation of diffuse granulomas in a plethora of organs, including the lungs and the hilar lymph nodes [18]. While the exact etiology of sarcoidosis remains unclear, several infectious agents are thought to display antigens that have the potential to initiate a cascade of inflammatory processes leading to granuloma formation [18]. Cardiac involvement is becoming more prevalent among patients of sarcoidosis and generally results in a poor prognosis [18]. Interestingly, 25% of sarcoidosis patients demonstrate cardiac involvement, which is associated with a 25% purported mortality rate in patients with symptomatic cardiac sarcoidosis [2]. Clinically, diagnosing sarcoidosis with cardiac involvement is extraordinarily onerous. Given this considerable challenge, CMR provides a diagnostic advantage in comparison to other imaging modalities [19]. CMR is noted to manifest sensitivity and specificity of 100% and 78%, respectively, in diagnosing cardiac involvement in patients with sarcoidosis and should thus be used as part of the initial diagnostic armamentarium in such patients [20]. 

While numerous imaging modalities for the detection of NICM are available, CMR is noted to be more sensitive, specific, while yielding timely diagnoses [18-20]. Pertinently, the specific patterns of LGE, in combination with clinical findings and meticulous cardiac evaluation, can elucidate the particular etiologies of nonischemic cardiomyopathy cases. The present study unequivocally demonstrates the utility of CMR imaging in yielding a timely diagnosis of a multitude of aforementioned nonischemic cardiomyopathies. Owing to its remarkable sensitivity and specificity, CMR should be considered a routine part of the initial investigations performed on patients with NICM.

## Conclusions

Nonischemic cardiomyopathies are a significant cause of cardiac morbidity and mortality. Due to the vast array of etiologies that can potentially culminate in the onset of nonischemic cardiomyopathy, CMR should be used as part of the initial diagnostic armamentarium in patients with nonischemic cardiac ailments. In addition to being exceedingly sensitive and specific, CMR can be employed for the detection of more infrequent cardiac pathologies including cardiac sarcoidosis and cardiac amyloidosis. 
